# Celebrating Rodolfo Margaria and applied physiology in Milan

**DOI:** 10.1113/EP093979

**Published:** 2026-08-13

**Authors:** Alberto E. Minetti, Gaspare Pavei, Damian M. Bailey

**Affiliations:** ^1^ Division of Physiology, Department of Pathophysiology and Transplantation University of Milan Milan Italy; ^2^ Neurovascular Research Laboratory, Faculty of Life Sciences and Education University of South Wales Pontypridd UK

This Editorial celebrates the convergence in Milan of three events of special significance to the Italian physiology community and to The Physiological Society: the unveiling of a Blue Plaque honouring Professor Rodolfo Margaria's seminal contributions; the opening of celebrations marking the 150th Anniversary of The Physiological Society; and the biennial conference Lavori in Corso (Work in Progress) 2026, a meeting dedicated to applied physiology that has recently expanded its international reach.

Introduced by The Physiological Society, Blue Plaques ‘honour outstanding physiologists who have contributed to the advancement of the discipline through their discoveries while leaving a legacy beyond their lifetime’ (The Physiological Society, 2026). They also create a lasting physical link between the scientist being celebrated and the place in which much of their most influential work was conceived and conducted. In this case, that place is the former Institute of Physiology at the University of Milan in Italy. It was therefore a fitting tribute to Italian physiology that, on 23 January 2026, the first international Blue Plaque awarded by The Physiological Society outside the UK and Ireland was unveiled in Milan (Figure 1).

**FIGURE 1:**
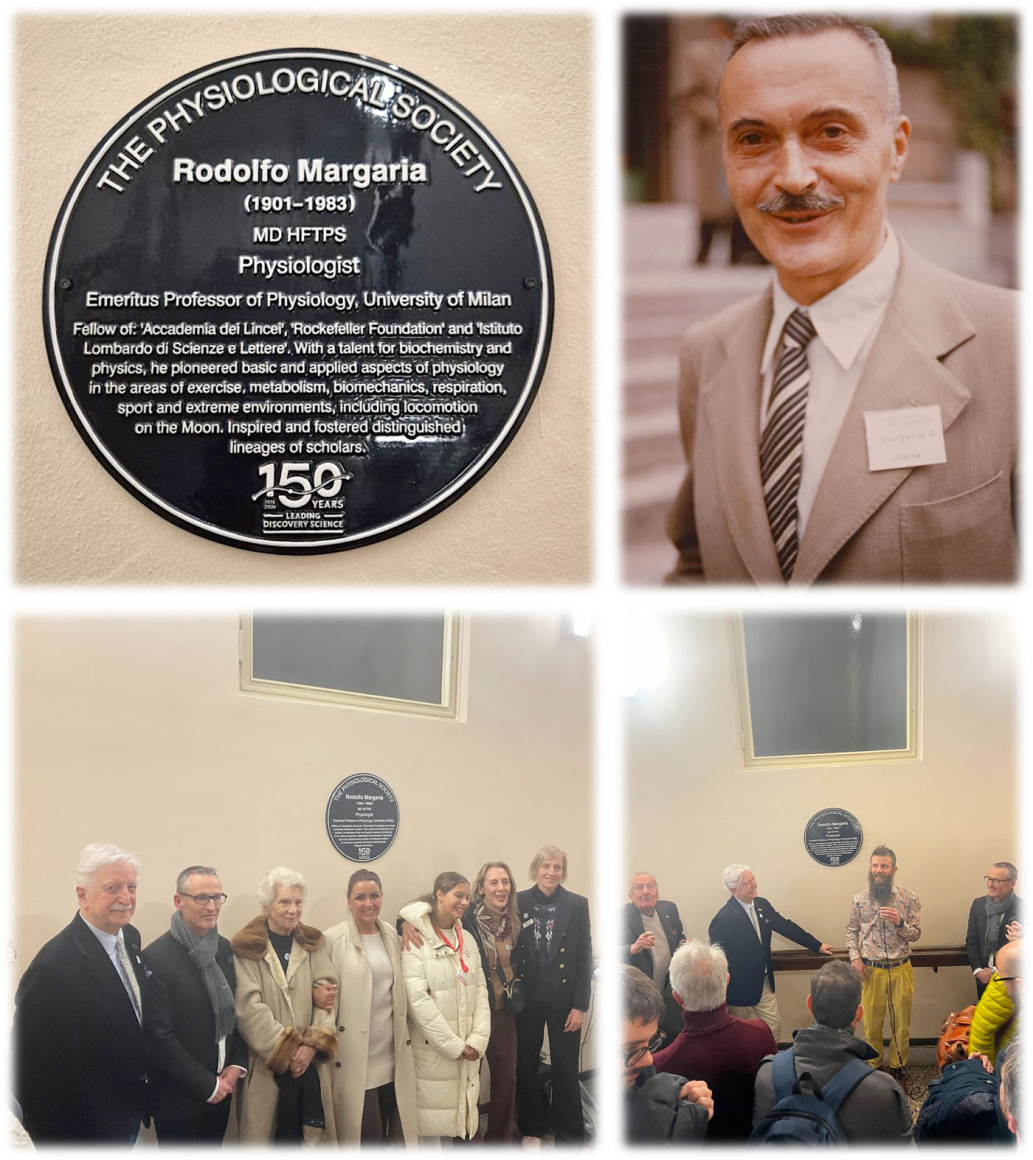
Honouring Professor Rodolfo Margaria at the Blue Plaque unveiling in Milan. The Blue Plaque awarded by The Physiological Society now hangs in the Pathophysiology and Transplantation Department at the University of Milan, in the building where Professor Rodolfo Margaria worked for more than four decades. The figure also shows Margaria (circa 1960), members of his family, including Dr Elsa Margaria MD and Architect Martina Margaria, and, right, Professor Pietro E. di Prampero, one of Margaria's former pupils and a pioneer in the field, with Associate Professor Gaspare Pavei. Together, they recalled Margaria's curiosity, humility, scientific rigour, intellectual generosity, commitment to mentorship, and enduring influence on the physiological community, which continues to shape and inspire physiologists today.

Rodolfo Margaria (1901–1983) obtained his medical degree in 1924 and his Teaching Habilitation (Docenza) in (Human) Experimental Physiology in 1928. His intellectual training was unusually broad, spanning chemistry, biochemistry, biophysics, thermodynamics and physiology. His academic career began in Ferrara, where he was appointed Professor of Physiology in 1934, before moving to Milan, where most of his academic activity was based between 1938 and 1972. Extended periods at leading international laboratories, including at University College London, Harvard University, the Kaiser Wilhelm Institute in Heidelberg and Yale University, allowed him to interact with leading figures in physiology, including A. V. Hill, himself later honoured with a Blue Plaque by The Physiological Society.

The first decades of the 20th century were decisive in shaping Margaria's scientific interests and his distinctively multidisciplinary vision of applied physiology. The term had already appeared in textbooks by Samson Wright in the UK and Best and Taylor in the USA (Tipton, 2016) and was defined by Dill (1939) as ‘muscular exercise being performed at the work or performance site, including fatigue and training, blood changes, effects of age, high environmental temperatures, and the influences of low and high partial pressures of oxygen’. Many of Margaria's own recurring themes, encompassing altitude, aviation, gravity, space, diving, swimming, walking, running, skiing, phonation, sport, ergonomics and ageing, map closely onto, and in some cases extend beyond, this early definition. They reveal a scientist willing to move across disciplinary boundaries while retaining a common physiological logic and methodology, including the use of nomograms in biomedicine as an analogue computational tool in a pre‐digital era.

Inspired, at least in spirit, by institutional models such as the Harvard Fatigue Laboratory, which Lawrence J. Henderson helped to establish in 1927 as a research laboratory able to attract commercial support while remaining outside the responsibility of a single department or college (Chapman, 1990), Margaria founded and directed the Centro Studi Fisiologia del Lavoro Muscolare of the Italian National Research Council between 1969 and 1974. From this Centre, and from other research facilities that he helped to establish, the ‘Milanese School of Physiology’ began to flourish, carried forwards by many of his former collaborators. These included Giovanni Cavagna (38 co‐authored publications), Paolo Cerretelli (24), Giorgio Torelli (19), Torquato Gualtierotti (16), Franco Saibene (14), Eloisa Milla (13) and Pietro E. di Prampero (11). Professor Margaria also actively attracted visiting scientists from abroad to work across the many laboratories he directed, thereby fostering a strong and enduring culture of international collaboration.

It is beyond the scope of this Editorial to catalogue the full breadth of Margaria's scientific advances. However, three areas stand out for brief mention. First, in muscle bioenergetics, he helped to define the three principal sources of energy during exercise (aerobic, anaerobic ‘lactic’ and anaerobic ‘alactic’), through his classic description of oxygen debt and its repayment (Margaria et al., 1933). Second, in respiratory physiology, his work extended from the role of carbonic anhydrase to estimates of respiratory efficiency based on the mechanical work of breathing (Margaria et al., 1960). Third, in the bioenergetics and biomechanics of locomotion, he provided an integrated account of the metabolic cost of walking and running across gradients and speeds, coupled with estimates of mechanical work and locomotor efficiency (Margaria, 1976). This thinking also anticipated the altered mechanics of walking on the Moon, including the likely preference for a bouncing gait, ∼5 years before the Apollo landing (Margaria & Cavagna, 1964). Owing to his vast scientific culture, integrated mechanical insight, enthusiasm, creativity and curiosity, Margaria was remembered by subsequent generations of collaborators as the ‘Grand Old Master’ (Ferretti, 2023).

Select examples of Margaria's seminal concepts, and their subsequent development by his successors, are summarised below.

Margaria's legacy continues to shape research into muscle and whole‐body bioenergetics, in addition to force and power production. The metabolic energy systems and their physiological limitations were explored in depth by his successors (di Prampero, 1981, 1986; Grassi et al., 2003). This work extended to studies performed in extreme environments, from high‐altitude experiments including the EvK2CNR Pyramid (Cerretelli, 1976; Grassi et al., 1996) to bed‐rest campaigns as an analogue of microgravity (Zuccarelli et al., 2021). These approaches helped to define the determinants and limits of human performance (Capelli et al., 2006; di Prampero, 2003). In parallel, studies of muscular force production moved from single‐fibre experiments (Cavagna et al., 1985) to NMR imaging and early ultrasonography, which revealed structural and mechanical changes during contraction (Narici et al., 1989, 1996). Bed‐rest campaigns and spaceflight studies further clarified the relative contribution of different factors to force loss and recovery (Antonutto et al., 1999; di Prampero & Narici, 2003; Monti et al., 2021). A consistent feature across these lines of work is the macro‐to‐micro approach inherited from Margaria: identifying physiological determinants at each biological level, probing the underlying mechanisms as techniques allow, but never losing sight of integrated whole‐body physiology (Zuccarelli et al., 2021).

Cavagna's mechanical engineering approach to biology extended Margaria's legacy by treating the musculoskeletal system as a thermodynamic machine. He identified the sources of external mechanical work during level walking and running (Cavagna et al., 1976). This work was later complemented by a combined metabolic and mechanical analysis of the optimal energetic cost of gradient locomotion at −18% (Minetti et al., 1993). This integrated framework was subsequently applied across a wide range of physiological settings. The economy of head‐supported load carriage was investigated in both arid (Heglund et al., 1995; Maloiy et al., 1986) and extreme‐altitude environments (Minetti et al., 2006), revealing the contribution of trunk oscillations to the metabolic cost of ascent.

A second enduring strand concerned locomotion in reduced gravity. Margaria and Cavagna proposed that the human locomotor apparatus, especially skeletal muscle, might be poorly suited to extraterrestrial environments with lower gravity than Earth (Margaria & Cavagna, 1964). This prediction reflected a broader physiological principle that muscle mechanics, neural control and the arrangement of body mass, joints and tendons have all been shaped by prolonged evolutionary conditioning under terrestrial gravity. Parabolic‐flight studies later supported this prediction (Cavagna et al., 1998), while theoretical analyses based on dynamic similarity and the Froude number helped to explain the decreased speed of optimum mechanical recovery and gait transition of pendular walking in reduced gravity (Minetti, 2001). Extending Margaria and Cavagna's curiosity for unusual and animal gaits (Cavagna et al., 1977), later work showed that skipping, a hybrid pendular–bouncing gait used by children and lemurs, was also spontaneously adopted by astronauts on the Moon (Minetti, 1998). This research line, now extending across almost six decades, remains active in Milan, where two low‐gravity simulation facilities, LOOP and M‐WALL, are permanently installed (Minetti et al., 2024).

The same comparative biomechanical perspective also yielded unexpected insights beyond human locomotion. Linked to Margaria's interest in the metabolic limits of intense exercise, this approach helped to explain the striking invariance of horse‐change station distance and gallop travel speed across pre‐industrial postal systems over the past three millennia (Minetti, 2003; Minetti et al., 1999). It also revealed the likely optimal mass of halteres, the hand‐held weights used in the ancient Olympic long jump, and their contribution to jump distance through a combination of experiments and computer modelling (Minetti & Ardigò, 2002).

Lavori in Corso (Work in Progress) is a biennial meeting held in Milan that draws inspiration from Margaria's celebrated ‘Wednesday afternoon’ discussions, when researchers gathered in his office to present and debate the progress of different lines of work within the Institute of Human Physiology. The format was revived ∼30 years later by Professor Franco Saibene (1932–2008) in the late 1990s, then again from 2011 by Alberto Minetti and Gaspare Pavei, who developed the meeting further while retaining its deliberately open and collegial spirit.

The aim is simply to bring together the applied physiology community in the historical Aula Magna of the Institute of Human Physiology to share ongoing work, early results and emerging ideas. The 2026 meeting, held in the presence of The Physiological Society and aligned with the Blue Plaque ceremony, marked an important evolution as the meeting switched to English and reached a wider international audience, attracting 120 participants from 27 institutions across six countries. The scientific programme reflected Margaria's enduring legacy: respiration, muscle, exercise, locomotion and extreme environments, with special attention to space physiology, a long‐standing theme in the Milanese tradition, alongside emerging areas, such as motor control and disease.

The enduring strength of the meeting lies in placing diverse expertise in the same room, from master's students to senior physiologists, with short presentations of work in progress followed by generous, constructive discussion. This format encourages critical engagement with methods, interpretation and future directions, while preserving the informality and intellectual curiosity that characterised Margaria's original gatherings. The meeting concluded with a farewell reception, where conversations continued around shared scientific legacies, new collaborations and future projects.

It was therefore especially fitting that The Physiological Society chose to begin its 150th Anniversary celebrations in Milan by honouring Rodolfo Margaria, a scientist whose work helped to define applied physiology as an integrative, outward‐looking discipline and whose legacy continues to animate the laboratories, meetings and communities that gather in his name. The plaque unveiling, twinned with the accompanying scientific meeting, represents one of many special events scheduled throughout the year to commemorate the Society's 150th Anniversary. Together, these events provide an opportunity not only to honour the pioneers who shaped physiology, but also to reaffirm the Society's continuing commitment to advancing the discipline, strengthening its community and inspiring future generations of physiologists.

## AUTHOR CONTRIBUTIONS

Damian M. Bailey and Alberto E. Minetti conceived the idea and wrote the first draft of the manuscript. Alberto E. Minetti, Gaspare Pavei and Damian M. Bailey approved the final version submitted for publication and agree to be accountable for all aspects of the work in ensuring that questions related to the accuracy or integrity of any part of the work are appropriately investigated and resolved. All persons designated as authors qualify for authorship, and all those who qualify for authorship are listed.

## CONFLICT OF INTEREST

Damian M. Bailey is Editor‐in‐Chief of *Experimental Physiology*.

## GENERATIVE AI STATEMENT

The authors confirm that no artificial intelligence tools, including large language models (LLMs), were used in the drafting or revision of this manuscript. All content was conceived, written and approved solely by the authors.

## Data Availability

The authors have nothing to report.
